# Unseen Wounds: Conceptualizing the Experience of Emotional Pain as a Distinct Neuropsychological Construct

**DOI:** 10.7759/cureus.105475

**Published:** 2026-03-19

**Authors:** Megha Agrawal, Gyan Vardhan, Yogesh Singh, Rajesh Kathrotia, Arun Goel

**Affiliations:** 1 Physiology, All India Institute of Medical Sciences, Jammu, Jammu, IND; 2 Pharmacology, All India Institute of Medical Sciences, Rishikesh, Rishikesh, IND; 3 Physiology, All India Institute of Medical Sciences, Rishikesh, Rishikesh, IND; 4 Physiology, All India Institute of Medical Sciences, Rajkot, Rajkot, IND

**Keywords:** acute pain, affective neuroscience, emotional distress, emotional pain, neurobiology of pain, psychological pain, social pain

## Abstract

Emotional pain, often triggered by adverse life events, varies widely in its expression--from feelings of “shattering” or “brokenness” to states of “numbing” or “blunting.” This raises the possibility that intense pain can be experienced without overt physical pathology. Once regarded as purely somatic, pain now recognizes an affective dimension that underscores the deep connection between mind and body. This conceptual article explores the nature of emotional pain, its distinctions from physical pain and examines whether it could be conceptualized as a distinct neuropsychological construct.

In the absence of observable external injury, emotional pain may be regarded as intrinsic--arising within the brain itself. Neuroimaging studies suggest that regions such as the anterior cingulate cortex and insula play key roles in emotional pain processing, in contrast to the somatosensory cortex that primarily mediates physical pain, although their modulatory pathways may partially overlap. Inflammatory mechanisms may also contribute to both emotional and physical pain; however, they are likely mediated through distinct pathways. Thus, the underlying mechanisms could involve neuropsychological, metabolic, and biochemical components that may manifest differently in individuals. These processes may cause agony or despair, impair quality of life, and in severe cases contribute to self-harm or the development of mental disorders. An exaggerated stress response may be key to the genesis of emotional pain, as evidenced by catecholamine-mediated Broken Heart Syndrome.

The World Health Organization (WHO) reports the prevalence of mental disorders in more than one billion people worldwide, with one in every hundred deaths due to suicide. In a world increasingly burdened by psychological morbidity, the psychophysiology of emotional pain remains an underexplored domain. Understanding its mechanisms could be vital for effective and timely intervention to mitigate neuropsychiatric morbidity.

## Editorial

Can pain exist beyond the physical?

Scientific research has traditionally focused on somatosensory pain-pain with observable physical markers-while largely overlooking emotional pain, despite its profound impact. Although mental stress is recognized as a contributing factor in 75-90% of diseases [1], emotionally driven pain is often dismissed as “imagined” or “all in the patient’s head” [2]. The World Health Organization reports that over one billion people worldwide live with mental health disorders, and suicide accounts for approximately one in every hundred deaths globally. It is estimated that twenty attempts occur for every death by suicide [3].

The question arises: Why are such experiences felt as real pain, even in the absence of physical injury? How can they lead to overwhelming suffering, despair, or even self-harm?

Individuals describe emotional pain in diverse ways, including feelings of being “broken,” “wounded,” or “scarred” [4] as well as experiences of emotional blunting, anhedonia, and profound numbness [5]. Although these descriptions borrow from physical injury metaphors, emotional pain is not merely symbolic [6]. Psychological distress can generate genuine physical symptoms and vice versa. Somatoform disorders, such as Conversion Disorder, Hypochondriasis, and Body Dysmorphic Disorder, described in the Diagnostic and Statistical Manual of Mental Disorders (DSM), Third Edition, Revised, illustrate how affective distress may manifest through physical symptoms, while similar interactions between emotional and somatic processes are also observed in conditions such as Irritable Bowel Syndrome and Chronic Fatigue Syndrome [7]. In the DSM-5, these conditions were reclassified as somatic symptoms and related disorders, emphasizing distressing symptoms and psychological responses rather than the absence of a medical explanation [8]. This raises important questions about why individuals become mentally overwhelmed and what neural mechanisms underlie such experiences.

As global awareness of mental health grows, it is essential to define emotional pain and consider whether it represents a distinct phenomenon. The terms “emotional” and “psychological” pain are often used interchangeably, though some researchers argue that “psychological pain” is broader, encompassing emotions, cognitive beliefs, and self-appraisal [9]. Cognitive modulation of pain involves regions such as the parietal cortex and insula as opposed to affective pain processing engaging the anterior cingulate cortex (ACC), prefrontal cortex, and periaqueductal gray [10]. Additionally, emotional pain overlaps conceptually with related affective states such as social pain, trauma, burnout, and depression. A defining feature, however, is the intense sense of inner brokenness or psychological agony that is not usually experienced in other mental states [11].

How is emotional pain different from physical pain?

Pain is traditionally defined as an unpleasant sensory and emotional experience associated with actual or potential tissue damage [12]. It is subjective, shaped by biological, psychological, social, and environmental factors, and not always easy to articulate [13]. In recognition of this complexity, the International Association for the Study of Pain (IASP) revised its definition to emphasize pain's multifaceted nature [14].

While physical pain often fades with time, emotional pain tends to linger in the mind, often reinforced by rumination, challenging the notion that pain arises solely from sensory input. Emotional pain--also termed psychological or mental pain--is defined as suffering that originates primarily from psychological causes rather than physical stimuli [15]. It can be triggered by emotionally distressing events even without physical harm [4] and is strongly linked to conditions such as depression, anxiety, psychosis, and suicide [16]. Despite these links, emotional pain remains poorly understood.

**Figure 1:**
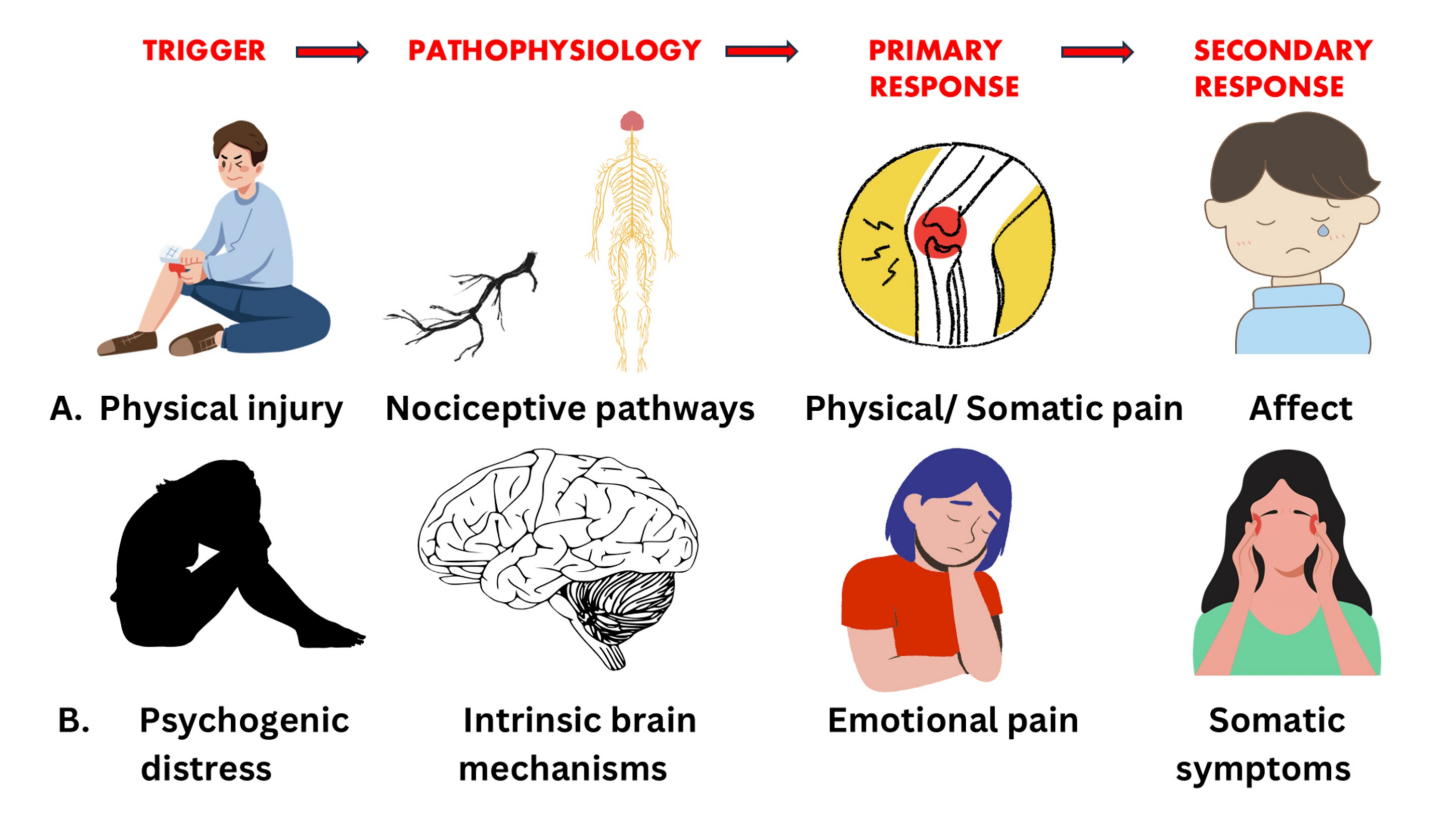
Conceptual model comparing (A) physical pain and (B) emotional pain. The diagram illustrates differences in origin, neural pathways, subjective experience, and clinical implications between the two constructs. Original artwork created using Canva (Sydney, Australia).

Modern perspectives distinguish psychogenic pain from nociception (Figure 1). While physical pain signals bodily threat or damage, emotional pain is characterized by themes of loss, hopelessness, and identity disruption [4]. Physical pain arises from peripheral tissue injury detected by nociceptors, whereas emotional pain is centrally mediated [17]. Clinical observations in chronic and terminal illness revealed forms of suffering that occurred without nociceptive input, often described as anguish rather than physical pain [18]. Neuroimaging studies indicate that emotional pain primarily activates the ACC and prefrontal cortex in contrast to physical pain, which engages somatosensory regions [19]. Social rejection, for example, consistently activates the ACC and anterior insula, regions associated with affective distress [20]. Myocardial stunning mediated by catecholamines released during hyperstimulation of the sympathetic nervous system in response to acute emotional stress is the most widely accepted mechanism underlying “Broken Heart Syndrome”, also known as “Stress cardiomyopathy” or “Takotsubo cardiomyopathy” [21]. Thus, an exaggerated stress response could be central to the genesis of emotional pain [22].

Inflammation may be common to both emotional and physical pain; however, the mechanisms initiating these processes may be distinct. Somatic pain is typically driven by peripheral tissue injury and nociceptor-mediated inflammatory processes that lead to central sensitization [17], whereas emotional pain is more closely associated with stress-induced neuroimmune activation influencing limbic and affective brain circuits [23]. Stress-evoked sterile inflammation has also been implicated in increasing vulnerability to mood disorders [22]. 

As emotional pain may lead to somatic symptoms and physical pain may have an affective component, the possibility of interconnected pathways cannot be ruled out [6,7] (Figure 1). Emerging evidence suggests partial overlap between the physical and emotional pain networks [6, 24], and neurobiological evidence indicates that social pain can be mitigated by analgesics such as acetaminophen, just as physical pain [25]. Pain modulation relies on supraspinal systems and endogenous opioids, as well as neurotransmitters such as norepinephrine, dopamine, and gamma-aminobutyric acid (GABA), which regulate pain intensity and emotional salience [26]. These systems likely influence both physical and emotional pain.

Challenges and future directions

An important question remains unresolved: can emotional pain arise as a purely intrinsic neuropsychological phenomenon, independent of external harm? How does it manifest subjectively as agony, numbness, or even physical/somatic symptoms without overt evidence of tissue damage? Furthermore, what distinguishes emotional pain from related states such as stress, trauma, or burnout? Conceptualizing emotional pain as a distinct clinical and psychological entity may provide a clearer foundation before investigating its underlying neurobiological mechanisms.

However, research in this area presents significant challenges. Much of the current understanding relies on self-report measures, which are inherently subjective and influenced by individual differences in perception and expression. Neuroimaging studies using techniques like functional magnetic resonance imaging (fMRI), while informative, often lack ecological validity and may not fully capture the complexity of lived emotional experiences. Additionally, individuals vary considerably in their thresholds and interpretations of pain, complicating efforts to establish standardized measures.

Future research should focus on defining what it is, identifying reliable biological markers or neural signatures of emotional pain and examining its potential neuroplastic effects over time. It may also be valuable to distinguish between acute and chronic forms of emotional pain and to explore their respective long-term impacts on mental health. A deeper and more integrative investigation of emotional pain could not only enhance prevention and treatment strategies but also transform broader conceptualizations of mental health across clinical and cultural contexts.

Conclusion

In conclusion, pain generated in the brain due to psychogenic distress is fundamentally different from physical pain. While physical pain typically arises from tissue injury and follows identifiable somatosensory pathways, emotional pain is rooted in psychological distress, involving distinct yet overlapping neural circuits and brain regions. Its presentation can vary widely, often without clear physical findings, making assessment more complex and subjective.

As intrinsic neural mechanisms of emotional pain become clearer, it would be easier to detect disturbances in emotional processing at an early stage. Further research could help formulating tools for assessment of these mental states in health care facilities, using an evidence-based approach. Targeted interventions could be started based on the severity of the symptoms. Understanding these nuances is essential for accurate diagnosis and effective intervention, as management must address the underlying psychological factors alongside the experience of pain itself. This could help improve the quality of life or reduce psychological morbidity for many affected individuals worldwide.
